# Multicharged Phthalocyanines as Selective Ligands for G-Quadruplex DNA Structures

**DOI:** 10.3390/molecules24040733

**Published:** 2019-02-18

**Authors:** Catarina I. V. Ramos, Susana P. Almeida, Leandro M. O. Lourenço, Patrícia M. R. Pereira, Rosa Fernandes, M. Amparo F. Faustino, João P. C. Tomé, Josué Carvalho, Carla Cruz, M. Graça P. M. S. Neves

**Affiliations:** 1 QOPNA & LAQV-REQUIMTE, Department of Chemistry, University of Aveiro, 3810-193 Aveiro, Portugal; susana.p.almeida@ua.pt (S.P.A.); leandrolourenco@ua.pt (L.M.O.L.); ribeirop@mskcc.org (P.M.R.P.); faustino@ua.pt (M.A.F.F.); 2 Coimbra Institute for Clinical and Biomedical Research (iCBR), Faculty of Medicine, University of Coimbra, 3000-548 Coimbra, Portugal; rcfernandes@fmed.uc.pt; 3 CNC.IBILI Consortium, University of Coimbra, 3000-548 Coimbra, Portugal; 4 CQE & Departamento de Engenharia Química, Instituto Superior Técnico, Universidade de Lisboa, Av. Rovisco Pais, n1, 1049-001 Lisboa, Portugal; jtome@tecnico.ulisboa.pt; 5 CICS-UBI—Centro de Investigação em Ciências da Saúde, Universidade da Beira Interior, Av. Infante D. Henrique, 6200-506 Covilhã, Portugal; josueocarvalho@gmail.com (J.C.); carlacruz@fcsaude.ubi.pt (C.C.)

**Keywords:** multicharged phthalocyanines, G-Quadruplexes, telomerase inhibition, selectivity, salmon sperm DNA, UV-Vis, hyperchromism, G4-FID, circular dichroism

## Abstract

The stabilization of G-Quadruplex DNA structures by ligands is a promising strategy for telomerase inhibition in cancer therapy since this enzyme is responsible for the unlimited proliferation of cancer cells. To assess the potential of a compound as a telomerase inhibitor, selectivity for quadruplex over duplex DNA is a fundamental attribute, as the drug must be able to recognize quadruplex DNA in the presence of a large amount of duplex DNA, in the cellular nucleus. By using different spectroscopic techniques, such as ultraviolet-visible, fluorescence and circular dichroism, this work evaluates the potential of a series of multicharged phthalocyanines, bearing four or eight positive charges, as G-Quadruplex stabilizing ligands. This work led us to conclude that the existence of a balance between the number and position of the positive charges in the phthalocyanine structure is a fundamental attribute for its selectivity for G-Quadruplex structures over duplex DNA structures. Two of the studied phthalocyanines, one with four peripheral positive charges (ZnPc1) and the other with less exposed eight positive charges (ZnPc4) showed high selectivity and affinity for G-Quadruplex over duplex DNA structures and were able to accumulate in the nucleus of UM-UC-3 bladder cancer cells.

## 1. Introduction

Telomeres are special structures localized at each end of chromosomes which contribute to chromosomal and genomic stability by protecting the end of chromosomes from enzymatic degradation [1,2,3,4]. Their main functions are to maintain the stability of the chromosome structure, to ensure that the genetic information is perfectly copied when the cell duplicates, and to prevent the junction between the ends of consecutive chromosomes which could lead to the degradation of deoxyribonucleic acid (DNA) or to the occurrence of genetic mutations which can culminate in the appearance of tumors [5].

In normal somatic cells, telomeres are shortened during DNA replication and eventually become too short to protect the chromosome, leading to cell senescence and death. Many cancer cells can counteract this process by increasing telomerase activity. Telomerase is a reverse transcriptase enzyme, overexpressed in a range of cancer cells [1,3] that allows continuous cell division without telomere shortening [2] and thus this enzyme is an attractive therapeutic target. The inhibition of its activity by the stabilization of the terminal region of the telomeres, namely of DNA secondary structures such as G-Quadruplexes (GQ), by adequate ligands (e.g., TMPyP, Braco-19, PIPER) has been considered a promising strategy to achieve antitumor activity [6,7,8,9,10].

**Figure 1:**
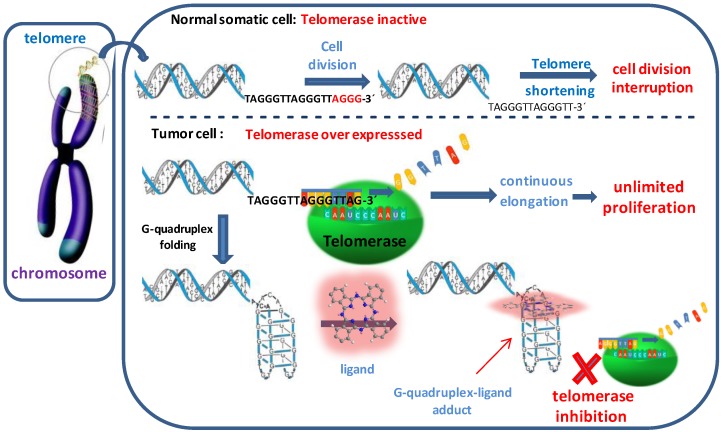
Schematic demonstration of telomerase inhibition by formation and stabilization of G-Quadruplex structures.

The indirect targeting of telomerase, through the binding of stabilizing compounds to GQ structures “locks” the telomeres in the quadruplex configuration and prevents telomere lengthening by telomerase [6,11] (Figure 1) since this enzyme only acts in single-stranded DNA and cannot recognize GQ as substrates [12].

These higher-order DNA structures are formed by G-quartets resulting from the self-assembly of four guanine (G) bases in a planar quadrangular arrangement via Hoogsteen hydrogen bonding. The subsequent stacking of the G-quartets on top of each other via π-π interactions give rise to different GQ conformations that are dependent on the number of DNA strands involved on the arrangement. These structures can involve one, two or four separate strands of DNA forming GQ with uni, bi or tetramolecular conformations. In the particular case of human telomeric DNA, tandem repeats of thymine (T), adenine (A) and guanine (G), in the sequence TTAGGG, (T_2_AG_3_), form unimolecular GQ at the end regions of chromosomes. The formation of GQ structures can also occur in physiological conditions in the presence of monovalent cations such as Na^+^ and K^+^, [13] and this is an important aspect considering the wide range of studies involving the investigation of G-Quadruplexes as targets for drug design [4,14,15,16,17].

In this context, several studies have shown that porphyrins and porphyrin analogues establish adduct formation with these GQ to inhibit telomerase activity [18,19,20,21,22,23,24,25,26]. A growing body of literature has shown that 5,10,15,20-tetrakis(1-methylpyridinium-4-yl)porphyrin (TMPyP) exhibits high affinity to GQ and thus it is a potential telomerase inhibitor. However, it is also recognized that this cationic and water soluble ligand has poor selectivity for GQ over duplex DNA structures [27,28,29,30].

Telomerase inhibitors should have higher selectivity for GQ DNA structures when compared with duplex DNA as the drug must be able to recognize quadruplex DNA in the presence of a large amount of duplex DNA, in the cellular nucleus. Competition between interactions with duplex versus GQ structures will reduce the availability of the ligand to bind to GQ, resulting in a reduction of its telomerase inhibitory function [31].

Along with porphyrins, phthalocyanines (Pcs) are emerging as GQ stabilizers and thus potential telomerase inhibitors [32,33,34]. These aromatic molecules hold a larger *π*-planar core than porphyrins that could be useful to increase their affinity and selectivity to GQ through π-π interactions [32]. Moreover, the presence of positive charges enabling the electrostatic interaction with DNA negatively charged backbone is also envisaged as an important feature for telomerase inhibition [35,36,37,38].

**Figure 2:**
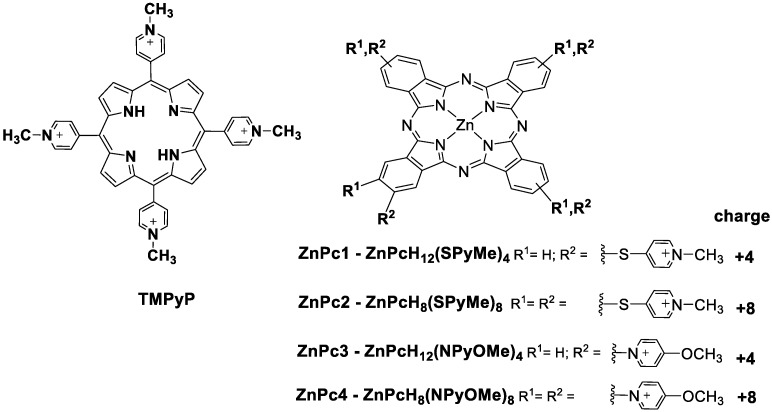
Structures of the studied TMPyP and thiopyridinium (ZnPc1, ZnPc2) and methoxypyridinium (ZnPc3, ZnPc4) phthalocyanines.

Within the framework of these criteria and following our interests in developing tetrapyrrolic macrocycles for biological applications [30,39,40,41,42,43,44,45,46,47,48,49], in this study we analyze if the cationic Pcs, ZnPc1, ZnPc2, ZnPc3, ZnPc4 bearing four or eight positively charged pyridine units (Figure 2) are able to stabilize telomeric DNA, namely GQ structures. This work examines not only the importance of the number of positive charges on the tetraazaisoindole macrocycle (i.e., ZnPc1 and ZnPc3 contain four positive charges, ZnPc2 and ZnPc4 contain eight positive charges), but also their location (i.e., ZnPc3 and ZnPc4 are inverted Pcs). We undertook this study in different DNA sequences (Table 1), namely the human telomeric repeat [50] (AG_3_(T_2_AG_3_)_3_) able to form a G-Quadruplex in unimolecular topology, the bimolecular *Oxytricha* repeat oligonucleotide [51] (G_4_T_4_G_4_)_2_ and the tetramolecular sequence (T_2_G_5_T)_4_. Two duplex DNA structures, a small (5GC) and a long chain DNA strand (salmon sperm), were also studied in order to compare the affinity and to evaluate the selectivity of the tested phthalocyanines for G-Quadruplex structures. The widely studied porphyrin, TMPyP, was also used as the standard.

Considering the spectroscopic features of the Pcs (a Soret band around 300 nm and two Q bands with high intensity between 600 and 700 nm) their interactions with DNA structures were investigated, by using different spectroscopic techniques such as UV-Visible (UV-Vis) spectroscopy, G-Quadruplex fluorescent intercalator displacement (G4-FID) assay and circular dichroism (CD) experiments. Moreover, it was verified by fluorescence microscopy if the most promising ligands were able to accumulate in cell nucleus of UM-UC-3 bladder cancer cells. This is an essential feature to consider these compounds as potential ligands for telomerase inhibition.

## 2. Results and Discussion

### 2.1. UV-Vis Spectroscopy

The UV-Vis spectroscopy is a very useful technique to analyze the interactions between a molecule and DNA. Besides, most of the laboratories have available spectrophotometers for routinely optical studies, this method is rapid, require small amounts of reagents and is non-destructive [18,19,52].

When a ligand interacts with DNA structures, a red shift (bathochromic effect) accompanied by intensity changes (hypochromic/hyperchromic effects) occur in their characteristic absorbance bands. The bathochromic effect is the result of a decrease in the π/π* transition energy due to the coupling of the π bonding orbital of the DNA base pairs with the empty π* antibonding orbital of the ligands. As a consequence of different type of interactions, different absorption profiles are expected in the UV-Vis region.

When an intercalative binding process occurs, typical values of hypochromicity (higher than 35%) and of bathochromicity (red shift, ∆λ > 15 nm) in the Soret band are expected; it is important to take in account that these values were determined for long pieces of duplex DNA where the end stacking is not significant [24,53]. Due to the less direct contact between π-systems, changes in the UV-Vis absorption spectra are less remarkable for groove binding or outside binding for which red shifts lesser than 8 nm have been described [54,55]. Thus, by analysing the batho- and the hypochromic effects on the obtained spectra, at the end of the titrations, it is possible to evaluate the affinity, the selectivity and to predict the type of interaction.

**Figure 3:**
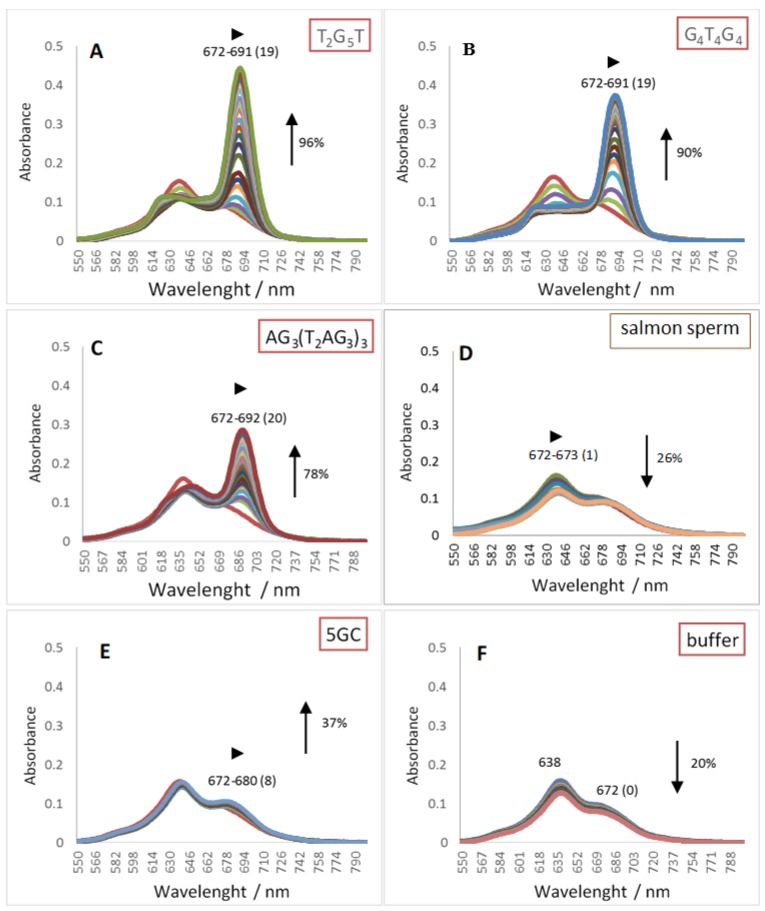
UV-Vis absorption spectra (550–800 nm) for the titrated solutions of ZnPc1 (2.0 μM) with (**A**) tetramolecular GQ, (**B**) bimolecular GQ, (**C**) unimolecular GQ, (**D**) salmon sperm, (**E**) 5GC, and (**F**) 20 mM PBS buffer with 100 mM KCl.

**Figure 4:**
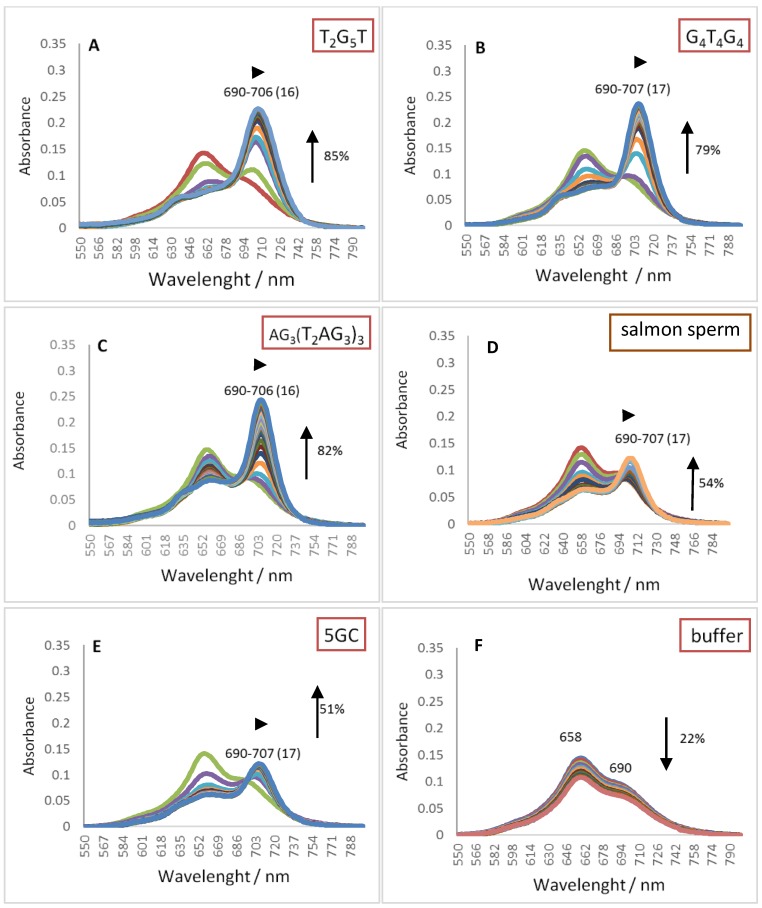
UV-Vis absorption spectra (550–800 nm) for the titrated solutions of ZnPc2 (2.0 μM) with (**A**) tetramolecular GQ, (**B**) bimolecular GQ, (**C**) unimolecular GQ, (**D**) salmon sperm, (**E**) 5GC, and (**F**) 20 mM PBS buffer with 100 mM KCl.

**Figure 5:**
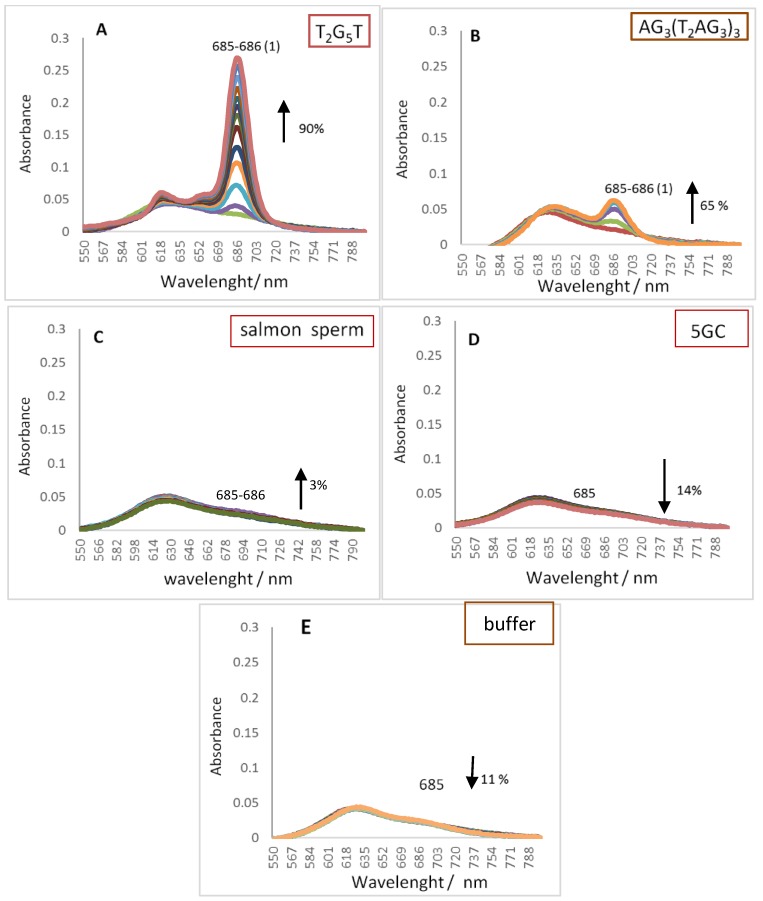
UV-Vis absorption spectra (550–800 nm) for the titrated solutions of ZnPc3 (2.0 μM) with (**A**) tetramolecular GQ, (**B**) unimolecular GQ, (**C**) salmon sperm, (**D**) 5GC, and (**E**) 20 mM PBS buffer with 100 mM KCl.

**Figure 6:**
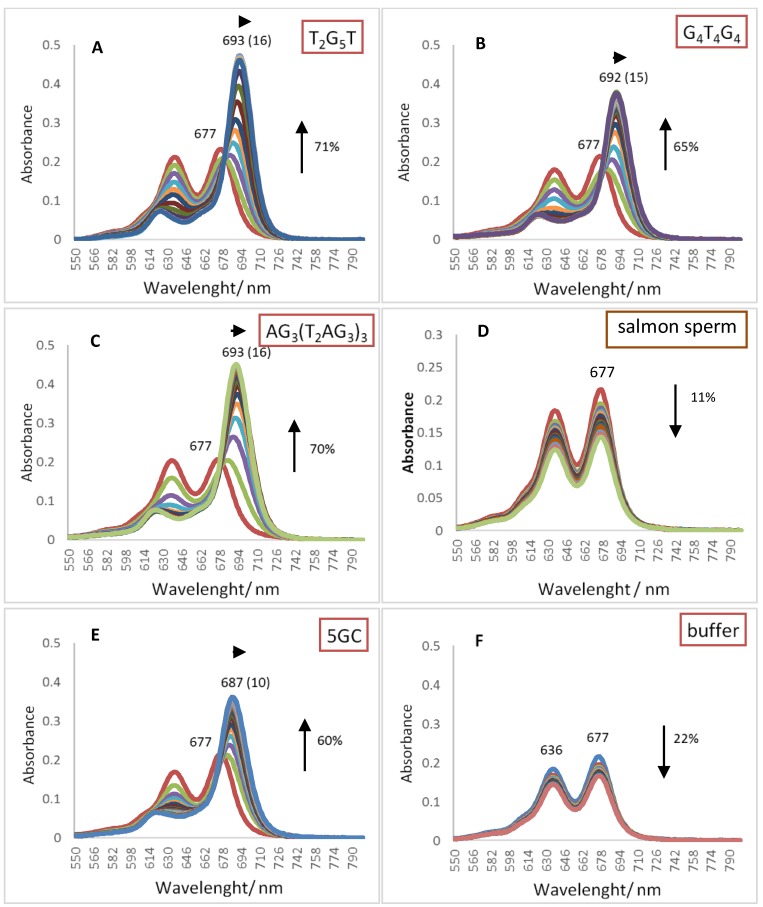
UV-Vis absorption spectra (550–800 nm) for the titrated solutions of **ZnPc4** (2.0 μM) with (**A**) tetramolecular GQ, (**B**) bimolecular GQ, (**C**) unimolecular GQ, (**D**) salmon sperm, (**E**) 5GC, and (**F**) 20 mM PBS buffer with 100 mM KCl.

Pcs electronic absorption spectra allow monitoring their interactions with oligonucleotide sequence namely GQ topologies using UV-Vis spectroscopy. The behavior of the three different DNA oligonucleotides, (T_2_G_5_T)_4_, (G_4_T_4_G_4_)_2_ and AG_3_(T_2_AG_3_)_3_, forming G-Quadruplex structures with different topologies, when interacting with the selected cationic phthalocyanines ZnPc1–4 was studied by UV-Vis titrations in the range of 350–800 nm. The titrations were performed by successive additions of the oligonucleotide in a phosphate buffer (PBS) to the phthalocyanine solution at an initial concentration of 2.0 μM, and were finished after four values of constant absorbance [53,56]. Control experiments were also performed by titration the Pc solutions with PBS that were then used to correct the absorbance values in the experimental data. Similar titrations were performed in the presence of the double chain DNA sequences 5GC and salmon sperm DNA. The interactions between ZnPcs1–4 and the different DNA sequences were carefully analyzed in the Q-band region (500–800 nm) and the obtained data for the selected Pcs are presented in Figure 3, Figure 4, Figure 5 and Figure 6. Table 2 summarizes the obtained results of bathochromism shifts and hypochromic or hyperchromic effects observed during titrations.

In order to validate our results and to confirm the reported data [27] about the non-selectivity of TMPyP to GQ topologies versus double chain DNA sequences, the assays were extended to this ligand. In these experiments, the main alterations occur in the Soret region (350–500 nm). The experiments showed similar bathochromism and hypochromic shifts for the double chain DNA and G-Quadruplex structures (Figure S1 and Table S1), with a slight increase in the bathochromism observed for the small double stranded DNA structure (∆λ = 11–16 nm for GQ and ∆λ = 18 nm for 5GC).

The obtained data for the tetracationic ZnPc1 (Figure 3 and Table 2, entry 1) shows that its interaction with GQ DNA structures (Figure 3A–C) results in alterations in the UV-Vis spectra that are different from the ones observed in the presence of duplex DNA (Figure 3D,E) structures.

In the presence of GQ structures, a large hyperchromism varying between 78 and 96% was accompanied by red shifts of *ca* 19 nm. In the presence of 5GC (Figure 3E), the hyperchromism (37%) and bathochromism (∆λ = 8 nm) were lower when compared with the ones observed in the presence of GQ DNA structures. The hypochromic effect (26%) detected in the presence of the longer double chain DNA sequence was accompanied by an insignificant alteration (∆λ = 1 nm) in the position of the Q band (Figure 3D). The hyperchromic effect observed when ZnPc1 is in the presence of GQ structures was already described for other multicharged Pc [51], but the percentage of hyperchromism was much less significant than the one observed with ZnPc1. It has been proposed that this hyperchromic effect results from the presence of GQ that prevents the occurrence of Pc aggregation probably due to incoming of GQ molecules between Pc molecules [57]. These macrocycles are described to be easily involved in stacking or aggregation processes, giving rise to dimers and high order aggregates due to the tendency of their hydrophobic skeleton to avoid the contact with water, so conditions that are able to prevent Pcs aggregation can improve their biological applications namely as photosensitizers since the production of reactive oxygen species (ROS) is really limited by aggregation [58].

The bathochromic deviations observed at the end of the different titrations are also promising since the values are considerable higher in the case of the GQ, when compared with the duplex structures. These data suggest that ZnPc1 interacts strongly with GQ structures.

The results obtained in the presence of the analogue bearing eight positive charges, ZnPc2, show a different interaction pattern (Figure 4 and Table 2, entry 2); the obtained data suggests similar affinity of ZnPc2 for all the studied DNA structures.

The considerable hyperchromism (between 79 and 85%) observed in the presence of GQ (Figure 4A–C) was also detected in the presence of duplex structures 5GC and salmon sperm (Figure 4D,E) although the variations were less impressive (*ca* 53%). All these alterations were accompanied by redshifts (∆λ = 16 or 17 nm) suggesting ZnPc2 high affinity for the generality of the DNA structures studied, from small to long duplex, from tetra to unimolecular GQ. This pattern, especially when compared with the selectivity behavior of the tetracationic analogue ZnPc1 points out that the eight positive charges present in ZnPc2 facilitates the interaction with the negative charged DNA backbone of all the studied DNA structures.

The inverted series of multicharged Pcs, the methoxypyridinium derivatives ZnPc3 and ZnPc4, containing positive charges not localized in the periphery of macrocycle, show that in the case of the tetracationic ZnPc3 (Figure 5 and Table 2, entry 3) the hyperchromism detected in the presence of the GQ topologies tetramolecular (T_2_G_5_T)_4_ and unimolecular (AG_3_(T_2_AG_3_)_3_) were accompanied by despicable red shifts of ∆λ = 1 nm (Figure 5A,B). Considering this behaviour the studies were not extended to the bimolecular GQ.

Insignificant bathochromic and hyperchromic deviations for salmon sperm (∆λ = 1 nm, 3%) and for 5GC duplex (hypochromism of 14%) were also observed in the DNA titrations with these structures. The significant hyperchromic effect observed in titrations with GQ are in accordance with the results previously obtained for the other Pc [57,58] and emphasize the importance of the presence of GQ in prevention of the occurrence of Pc aggregation processes probably due to incoming of GQ molecules between Pc molecules.

The analogous inverted Pc containing eight positive charges, ZnPc4, exhibits alterations in the UV-Vis spectra that suggest its high affinity for GQ DNA arrangements (Figure 6 and Table 2).

The analysis of the obtained data show that the hyperchromism detected in GQ and 5GC DNA structures (the range of 60–71%) were accompanied with red shifts of *ca* 15 nm only in the presence of GQ topologies. These data suggest that ZnPc4 presents also high affinity for all the GQ structures when compared to the duplex ones. Probably, the less peripheral location of the eight charges in the structure of the octa-substituted methoxypyridinium ZnPc4 is responsible by its higher selectivity to GQ when compared with the other eight-charged Pc, ZnPc2. The non-selective affinity of ZnPc2 for all DNA structures (GQ and duplex) can be related to the higher number of electrostatic interactions between the negative charges of DNA backbone and the more exposed positive charges of this specific Pc.

The UV-Vis titrations were also employed to determine the apparent equilibrium dissociation constants (KD) between the Pcs and the unimolecular GQ, AG_3_(T_2_AG_3_)_3_, and the double stranded 5GC. The results are presented in Table 3. Similar to the results obtained from the fluorimetric titrations (Section 2.2) the ligand ZnPc4 has the highest affinity towards AG_3_(T_2_AG_3_)_3_. In the case of 5GC, the KD values obtained are about 10^−4^–10^−5^ M higher than those obtained for the unimolecular GQ AG_3_(T_2_AG_3_)_3_, indicating weaker binding of all ligands to double stranded 5GC.

The selectivity of ligands towards GQ over duplex structures were also confirmed with the results obtained when the titrations were performed in the presence of a longer duplex DNA structure, from salmon sperm (Figure 3D, Figure 4D, Figure 5C and Figure 6C, and Table 2). When this long duplex DNA sequence was studied in the presence of ZnPc1 and ZnPc4, the lower bathochromic deviation observed (∆λ = 1 and 4 nm, respectively) were accompanied by a hypochromism effect. These results emphasize the affinity and selectivity of ZnPc1 and ZnPc4 to GQ structures. In contrast, ZnPc2 and TMPyP showed high bathochromic values (∆λ = 17 and 13 nm, respectively) confirming their high affinity for both type of DNA structures and consequently low selectivity.

**Figure 7:**
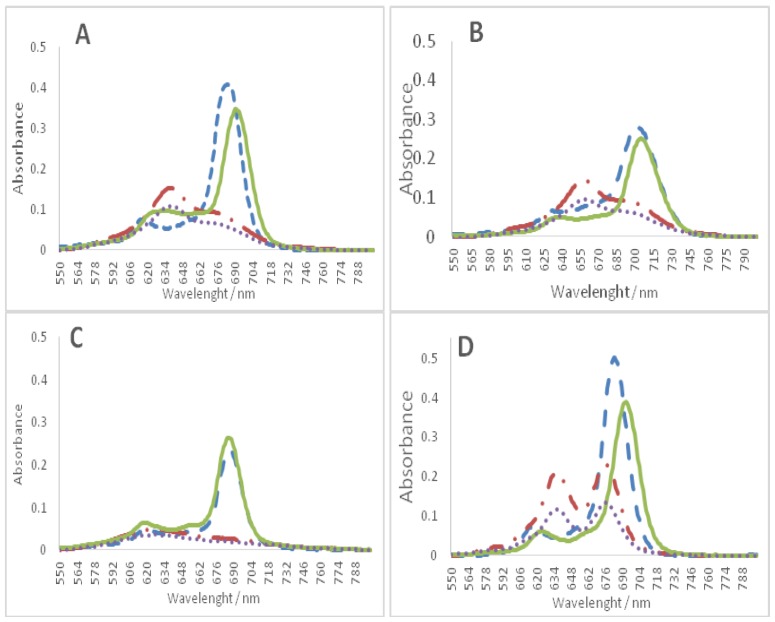
UV-Vis spectra of Pcs (2.0 μM) in DMSO (**---**), in PBS (**- . -**), and PBS + 200 μL PBS (.....), PBS + 200 μL GQ (T_2_G_5_T)_4_ (**—**); (**A**) ZnPc1, (**B**) ZnPc2, (**C**) ZnPc3, (**D**) ZnPc4.

Considering that in the titrations of the tetra-substituted methoxypyridinium ZnPc3, the hyperchromism of the Q band was not accompanied by a red shift, we decided to confirm that in these titrations the observed hyperchromism effects were related with disaggregation effects promoted by GQ or duplex structures and the red shifts with the affinity of the Pc to DNA structures. Therefore, the spectra of the studied ZnPcs1–4 were obtained in DMSO and compared with the previous spectra obtained in PBS and also with the ones obtained in the end of the titration with the tetramolecular GQ (T_2_G_5_T)_4_ oligonucleotide (Figure 7).

The obtained results are consistent with the hypothesis that the hyperchromism observed during the titration of ZnPc3 (Figure 7C) with the oligonucleotides is mainly due to a disaggregation process since the wavelength absorption maximum obtained in DMSO is the same to the one obtained at the end of titrations with GQ (Table S2). These results suggest that ZnPc3 does not interact with GQ.

The spectra of the other three Pcs (Figure 7A,B,D) show a different profile since red shifts of 4 to 10 nm, from the maxima obtained in DMSO, were detected; the highest red shift corresponding to ZnPc4. Considering that these red shifts are accompanied with hypochromism, this data could be interpreted as the hypochromic behavior observed for TMPyP. Thus, these results point out that ZnPc1 and ZnPc4 present high affinity and selectivity for GQ, while ZnPc2 shows a moderate interaction with the selected GQ DNA structure.

The hyperchromic effect observed for all Pcs is highly indicative that disaggregation occurs as a consequence of the GQ presence, and the red shit results from the interaction with GQ DNA structures. Considering the described behavior of ZnPc3 no further studies were performed with this ligand.

### 2.2. Fluorescence Experiments

Another well-established method to evaluate and confirm the affinity of a ligand for GQ and its selectivity for GQ towards duplex DNA is the G-Quadruplex fluorescent intercalator displacement (G4-FID) assay [18]. This assay is based on the loss of fluorescence of a probe like thiazole orange (TO) as a result of its displacement from DNA by a ligand [10,21]. The concentration of the ligands required to decrease the fluorescence of the probe (TO) by 50%, is noted by DC_50_.

**Figure 8:**
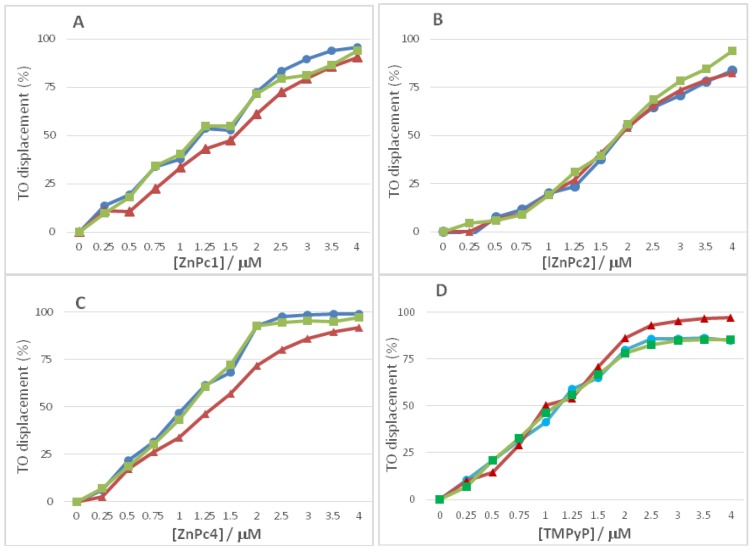
G4-FID assay performed in PBS at 25 °C with (T_2_G_5_T)_4_ (green squares), (AG_3_(T_2_AG_3_)_3_) (blue circles), duplex (5GC) (red triangles) and (**A**) ZnPc1, (**B**) ZnPc2, (**C**) ZnPc4, (**D**) TMPyP.

In order to validate the previous UV-Vis data, the ability of ZnPc1, ZnPc2, ZnPc4 and of TMPyP to displace TO from GQ structures (T_2_G_5_T)_4_ and (AG_3_(T_2_AG_3_)_3_) and also from the duplex 5GC was evaluated by fluorescence spectroscopy (Figure 8 and Table 4).

The obtained data from these G4-FID assays are in agreement with the results obtained from UV-Vis studies. ZnPc1 (Figure 8A) and ZnPc4 (Figure 8C) present the lower values of concentration to displace 50% of TO from the GQ-TO adducts when compared with the obtained values for 5GC-TO adducts. In the UV-Vis studies, ZnPc2 showed high affinity for GQ structures and also for duplex ones, and this low selectivity is corroborated by the similar concentrations obtained to displace 50% of TO from all the studied DNA structures (Figure 8B). A similar trend was observed for TMPyP (Figure 8D) which was consonant with its low selectivity for G-Quadruplexes versus DNA duplexes. 

**Figure 9:**
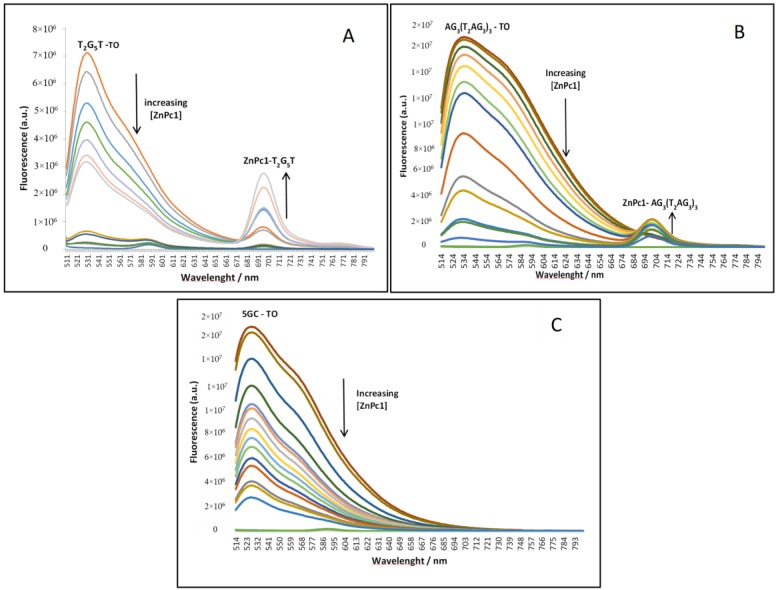
Fluorescence spectra obtained in PBS at 25 °C for ZnPc1 in the concentration range of 0–4.0 uM with (**A**) tetramolecular GQ (T_2_G_5_T)_4_, (**B**) unimolecular GQ AG_3_(T_2_AG_3_)_3_, and (**C**) duplex sequence 5GC.

The addition of increasing amounts of Pcs to the fluorescent solution containing the GQ and the TO probe resulted in a decrease in the GQ-TO band emission. Interestingly, along with this decrease an increase was observed on an emission band at *ca* 700 nm that probably corresponds to the formation of the adduct Pc-GQ. In the obtained spectra for 5GC no emission band was observed in the same region. This behavior is exemplified for ZnPc1 with the quadruplexes (T_2_G_5_T)_4_ (Figure 9A) and AG_3_(T_2_AG_3_)_3_ (Figure 9B), and also with the duplex 5GC (Figure 9C).

Similar behavior was observed for ZnPc4 (data not shown). These results are also in agreement with the previous ones, demonstrating that ZnPc1 and ZnPc4 present high affinity and selectivity towards GQ structures.

**Figure 10:**
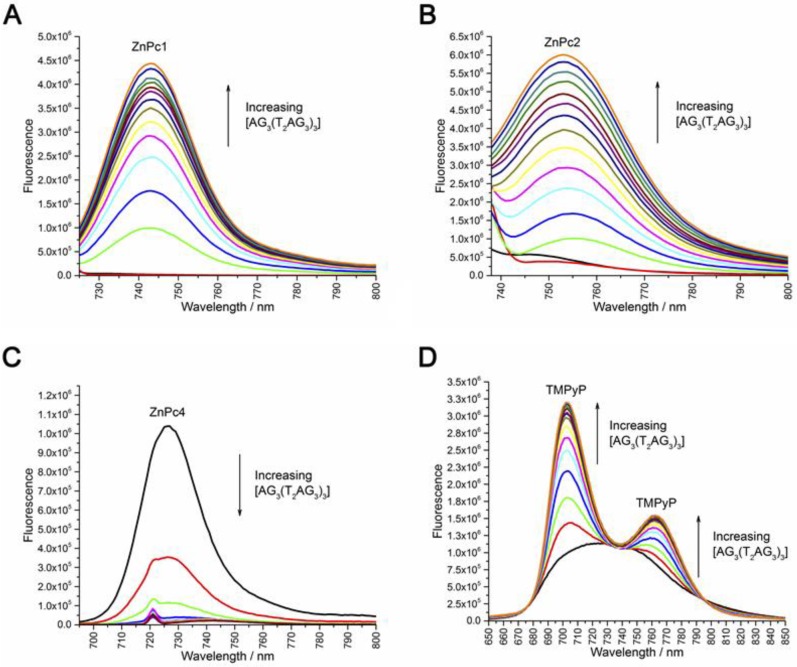
Fluorescence emission spectra of (**A**) ZnPc1, (**B**) ZnPc2, (**C**) ZnPc4 and (**D**) TMPyP with increasing concentration of unimolecular GQ AG_3_(T_2_AG_3_)_3_.

Additionally, fluorimetric titrations using the ligands intrinsic fluorescence were employed to determine the apparent equilibrium dissociation constants (*K_D_*) for the ligand′s interaction with (AG_3_(T_2_AG_3_)_3_) GQ. Interestingly, both ZnPc1 and ZnPc2 exhibited “turn-on” fluorescence upon titration with pre-folded (AG_3_(T_2_AG_3_)_3_) (Figure 10A,B, Figure S2A,B), presenting broad emission bands centered at 743 and 753 nm, respectively. This confirms the appearance of a fluorescence band during FID experiments being due to the formation of the adduct Pc-GQ. Similar effect has already been observed for similar phthalocyanine ligands upon interaction with c-MYC GQ [59]. The analysis of the fluorescence data using Hill saturation binding model revealed *K_D_* values of 3.6 × 10^−7^ and 5.4 × 10^−7^ M for ZnPc1/GQ and ZnPc2/GQ complexes, respectively. On the contrary, ZnPc4 exhibited fluorescence emission (λ_em_ = 726 nm) in the absence of GQ DNA but upon (AG_3_(T_2_AG_3_)_3_) titration a strong quenching of the ligand’s fluorescence was observed, indicating a different mode of binding (Figure 10C, Figure S2C). The obtained *K_D_* of 7.4 × 10^−8^ M indicates that ZnPc4 has higher affinity towards (AG_3_(T_2_AG_3_)_3_), even when compared to the well-studied porphyrin TMPyP which presented a 4-fold higher *K_D_* value 2.9 × 10^−7^ M (Figure 10D, Figure S2D). TMPyP exhibited a behavior similar to that reported in the literature with the appearance of two emission bands upon GQ titration [60]. The Hill coefficients (*n*) of ≈2 obtained for all ligands, suggest a ligand:GQ binding stoichiometry of 2:1, most likely by end-stacking interactions with the top and bottom tetrads of the GQ.

### 2.3. Circular Dichroism (CD)

Circular dichroism (CD) spectroscopy is a fast and simple methodology that provides rapid and valuable information about stabilization (or not) of a GQ structure by a ligand, and can be used at low or high oligonucleotide concentrations and for short or long DNA sequences.

CD spectroscopy can also be used to perform melting experiments and to determine melting temperature (Tm) of GQ, folding in different topologies, and in the presence of binding ligands. The difference between the obtained Tm for the GQ before and after the ligand addition is indicative of the stabilizing effect induced, in GQ structure, by the presence of a ligand.

**Figure 11:**
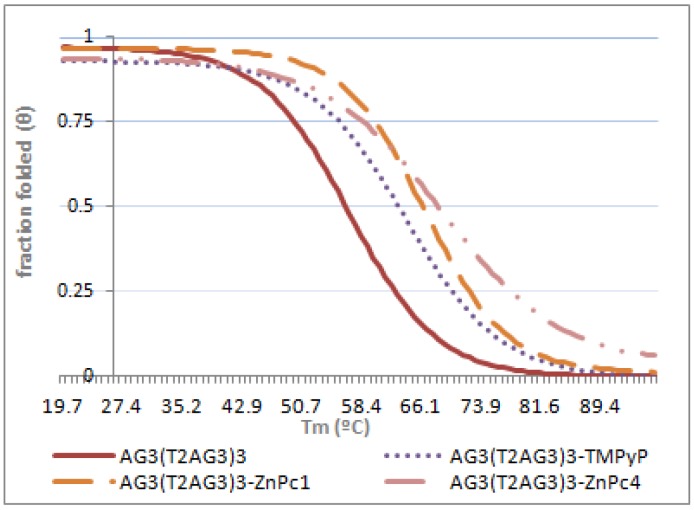
CD melting profiles obtained for GQ unimolecular AG_3_(T_2_AG_3_)_3_ in the absence or in the presence of ZnPc1, ZnPc4 and TMPyP.

The melting profiles obtained for the most promising ligands ZnPc1, ZnPc4 and also for TMPyP for comparison, were only performed in the presence of the human telomeric sequence AG_3_(T_2_AG_3_)_3_ (GQ unimolecular). The obtained data (Figure 11 and Table 5) show that, in general, the selected Pcs have a stabilizing effect in the GQ unimolecular structure, the Δ*T*_m_ being higher for Pc when compared with TMPyP.

The slight difference of 2 °C observed in the melting temperature of ZnPc4 when compared with ZnPc1, could be due to the higher number and position of charges present in the structure of ZnPc4. These results agree with the obtained equilibrium dissociation constants as ZnPc4 presented the lower *K_D_* value and also the higher Δ*T*_m_, demonstrating the higher affinity towards AG_3_(T_2_AG_3_)_3_ GQ.

Based on the fact that the characteristics CD spectral features for different folding topologies of GQ are already described, [61,62,63,64] the acquisition of the CD spectra provides important data, not only about the formation of proposed DNA structures but also about conformational changes induced by the presence of ligands. It is noteworthy that CD results cannot be used to unambiguously determine the type of quadruplex structure adopted by DNA, but it can be used to limit the number of possibilities [65]. 

The CD spectra of the unimolecular GQ AG_3_(T_2_AG_3_)_3_ in the absence and in the presence of increasing concentrations of the two most promising Pcs, ZnPc1 and ZnPc4, are shown in figures S3A and S3B, respectively. From these CD spectra, it was possible to demonstrate that the GQ folds correctly [61,63,65]. In the obtained CD spectrum for the unimolecular GQ (Figure S2), the positive band at 290 nm, with a shoulder at 270 nm and a negative band at 235 nm are consistent with a GQ structure in a hybrid type conformation [63,64]. Upon titration with ZnPc1, no overall changes in the general pattern of the CD spectra were detected (Figure S3A), despite a slight decrease in ellipticity which may be attributed to ligand binding [54,64]. In the case of ZnPc4, a structural interconversion was observed at 2 molar equivalents (Figure S3B), as shown by the positive band at around 265 nm and negative band around 240 nm, which is indicative of a parallel GQ conformation [66]. The 2:1 complex showed to be more stable than the hybrid type-bound ZnPc4, as no unfolded state was observed at 100 °C which gives a Δ*T*_m_ > 30 °C. Regarding TMPyP, upon ligand binding a deep conversion of the general pattern was observed (Figure S3C), in agreement with the previously reported in the literature [67]. Additional CD spectra of the duplex oligonucleotide 5GC in the presence and absence of ZnPc1 and ZnPc4 were acquired. For the duplex structure, the expected positive band in the range of 260–280 nm and the negative band at *ca* 245 nm were observed (Figure S4).

### 2.4. Phtalocyanine Nuclear Uptake and Toxicity in Bladder Cancer Cells

To validate the potential of Pc as ligands of G-Quadruplex DNA structures, we evaluated the ability of the most promising ligands to accumulate in the nucleus of UM-UC-3 bladder cancer cells. In vitro studies were performed with ZnPc1 and ZnPc4 since these ligands demonstrated high ability to bind GQ DNA structures as determined in our spectroscopy studies.

**Figure 12:**
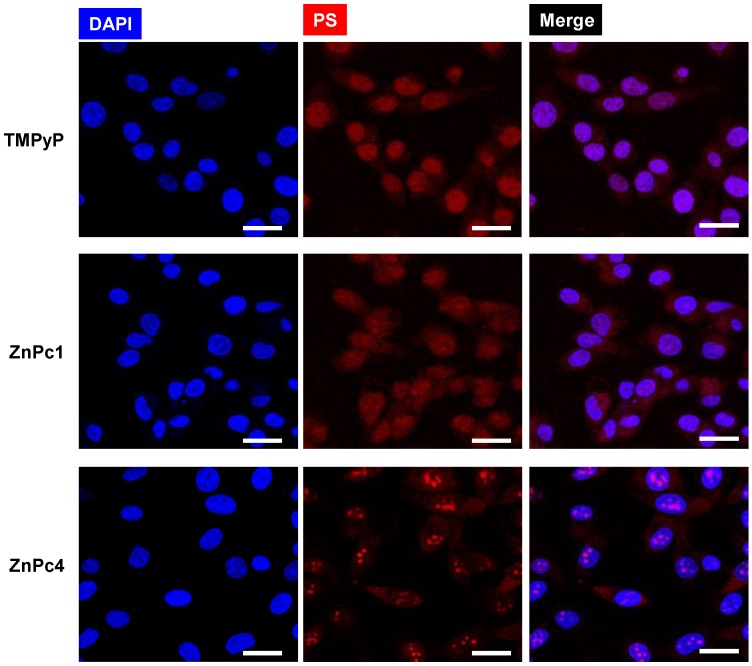
Representative fluorescence images of UM-UC-3 bladder cancer cells incubated with the ligands (red) TMPyP (30 μM), ZnPc1 (20 μM) or ZnPc4 (45 μM) for 48 h of incubation. DAPI is staining the nucleus. Scale bars 20 μM.

The internalization of Pc in UM-UC-3 bladder cancer cells was evaluated by fluorescence microscopy after cells incubation with TMPyP, ZnPc1 and ZnPc4 during 48 h (Figure 12). The merged blue [representing nucleus dye 4′,6-diamidino-2-phenylindole (DAPI)] and red (representing Pc) channels, fluorescence microscopy studies suggest that TMPyP, ZnPc1 and ZnPc4 accumulate in the nucleus of UM-UC-3 bladder cancer cells.

To investigate the antitumor potential of ZnPc1 and ZnPc4, MTT (3-[4,5-dimethylthiazol-2-yl]-2,5 diphenyl tetrazolium bromide) assay was performed to determine their cytotoxicity against the UM-UC-3 bladder cancer cells. Table 6 shows the IC_50_ (the concentration of the ligand required to reduce the cell viability by 50%) of ZnPc1, ZnPc4, and TMPyP after 48 h treatment. The IC50 of ZnPc1 is significantly lower than that of ZnPc4 or TMPyP, indicating a higher toxicity.

It is interesting to note that, although the IC_50_ values obtained for TMPyP was lower than the one of ZnPc4, the ligand concentrations used to evaluate the internalization of these Pcs in UM-UC-3 bladder cancer cells were lower than the IC_50_ value of TMPyP reference.

## 3. Materials and Methods

### 3.1. Chemicals

The chemicals were purchased as analytical grade and used without further purification and, when required, the solutions were prepared in MiliQ water. 5,10,15,20-Tetrakis- (1-methylpyridinium-4-yl)porphyrin tosylate (TMPyP) was purchased from Sigma Aldrich (Aldrich, Steinheim, Germany), and the lyophilized DNA oligonucleotides were purchased from Thermo Fisher Scientific (Waltham, MA, USA). The phthalocyanines were synthesized and characterized as previously described [45,46]. The molar extinction coefficients of the Pcs were determined in PBS (Table S3). For TMPyP and oligonucleotides the provided molar extinction coefficient values were considered. Stock solutions of the phthalocyanines were prepared in DMSO and stored at 4 °C. Before each assay an aliquot of the stock solution was diluted to a final concentration of 2.0 μM in PBS buffer.

### 3.2. Preparation of DNA Structures (Double Chain and G-Quadruplexes)

A PBS solution, containing 20 mM of phosphate buffer (10 mL of KH_2_PO_4_ 1 M, and 200 μL of K_2_HPO_4_ 1 M), 0.1 mM of ethylenediaminetetraacetic acid, and 100 mM of KCl was prepared with pH adjusted to 6.8. The PBS solution was used as the solvent for oligonucleotide solutions. After solubilisation in PBS each oligonucleotide was heated up to 85 °C for 10 min and left to cool overnight to assure the correct folding into double chain or GQ structures. The solutions were stored at −20 °C.

### 3.3. Methods

#### 3.3.1. UV-Vis Spectroscopy

UV-Vis absorption spectra were recorded in a UV-2501-PC spectrophotometer (Shimadzu Corporation, Kyoto, Japan) in the range between 350–800 nm, using reduced quartz cuvette of 1 cm length at controlled temperature (25 °C) by using a Compatible Control CC1 (Huber, Huntersville, NC, USA). During the spectroscopic titrations, all the compounds were dissolved in PBS to mimic the physiological conditions. Titrations were performed by successive additions of oligonucleotide solutions to 1 mL solution of phthalocyanine with the initial concentration of 2 μM, in the range of 350–800 nm. According to the literature, titrations were finished after 3–5 values of constant absorbance [68]. The spectra were mathematically corrected for the dilution effect and to ensure the reproducibility of results, all the experiments were performed in triplicate. The apparent equilibrium dissociation constants (KD) were determine using the Hypspec program.

The percentage of hypochromicity of the absorption band was calculated using the following equation %hypochromicity = [(ε_free_ − ε_bound_)/ε_free_] × 100, where ε_bound_ was calculated using the Beer’s Law (ε_bound_ = A_bound_/C_bound_) and ε_free_ are the extinction coefficient values reported in the literature for TMPyP ε_420_ = 226,000 M^−1^ cm^−1^and experimentally obtained, in PBS, in the case of Pcs, namely, ZnPc1 ε_638_ = 78,217 M^−1^ cm^−1^, ZnPc2 ε_658_ = 70,808 M^−1^ cm^−1^, ZnPc3 ε_628_ = 23,956 M^−1^ cm^−1^ and ZnPc4 ε_677_ = 106,299 M^−1^ cm^−1^.

#### 3.3.2. Fluorescence Spectroscopy

Stock solutions of 35 μM of TO and 10 μM of the different oligonucleotides were prepared. The previous solutions were mixed to obtain the desired TO-oligonucleotide solution and after 10 min of orbital shaking at 500 rpm, the fluorescence was measured in a Fluoromax-3 spectrofluorometer (Horiba, Kyoto, Japan), using excitation wavelength at 485 nm and emission range of 510–750 nm. Excitation and emission slits were set at 10 nm. The obtained fluorescence data were considered as *F*_*A*0_. Solutions with increasing concentrations (0 to 4 μM) of each ligand were prepared and added to the TO-Oligonucleotide ones and the fluorescence was measured using the same experimental parameters. The percentage of displacement, DC_50_ was calculated using the following equation:

(1) $$DC_{50}=100-\frac{F_{A}}{F_{A0}}\times 100$$

where $F_{A}=F-F_{H2O}$ and $F_{A0}=F_{0}-F_{H2O}$, *F* is the fluorescence intensity of each sample, *F*_*H*2*O*_ the fluorescence intensity of mili-Q and *F*_0_ the fluorescence from the fluorescent probe bound to DNA without added ligand.

For fluorescence titrations, spectra were recorded with a Horiba Floromax-4 spectrofluorometer. Ligands were excited at the wavelength of maximum absorption and fluorescence emission collected between 650–850 nm at 25 °C using a quartz cuvette with a path length of 1 cm containing 1 μM ligand solution. Excitation and emission slits were fixed at 5 nm. The association between GQ or and ligands was assessed by titrating the oligonucleotide (0 to 3 μM) and measuring the change in fluorescence. Data was converted into fraction of bound ligand (α) plots using the following equation:

(2) $$\alpha =\frac{I-I_{\lambda }^{free}}{I_{\lambda }^{bound}-I_{\lambda }^{free}}$$

where *I* is the fluorescence intensity each ligand:GQ ratio and *I_free_* and *I_bound_* are the fluorescence intensity of the free and fully bound ligand, respectively. Data points were then fitted to a hyperbolic function (OriginPro 8, OriginLab, Northampton, MA, USA) and *K_D_* values were determined from the following saturation binding model:

(3) $$\alpha =\frac{{\left[DNA\right]}^{h}}{K_{D}+{\left[DNA\right]}^{h}}$$

where *α* is the fraction of ligand bound, [*DNA*] is the concentration of the DNA and *h* is the Hill constant which describes cooperativity of ligand binding.

#### 3.3.3. Circular Dichroism

CD spectra were either acquired using J-1500 or J-815 CD spectrophotometers (Jasco, Pfungstadt, Deutschland) equipped with temperature controller Jasco PTC-517. The instrument was purged with pure nitrogen gas before analysis. For CD melting experiments, the solutions containing the DNA structures and the ligands (6 μM) were previously prepared, by heating the solution at 85 °C for 10 min and left to cool overnight. The CD melting spectra were obtained in the temperature range of 20–100 °C by monitoring the ellipticity at the characteristic wavelength of the hybrid type G-Quadruplex, 290 nm. Following the described literature [66], the obtained data were converted into fraction folded (θ) plots using the equation below, where CD is the ellipticity of the monitored wavelength at each temperature, and *CD^max^* is the highest ellipticity and *CD^min^* being the lowest ellipticity:

(4) $$\theta =\frac{CD-CD^{min}}{CD^{max}-CD^{min}}$$

The Origin 8.0 software was used to perform sigmoidal curve fitting, using the Boltzmann function. The melting temperatures were then obtained. For CD titrations, the spectra were obtained in the absence or presence of increasing concentration of the ligands between the wavelength range of 220–320 nm, using quartz cells of 1 cm length, and a bandwidth of 1 nm, three accumulations and a data integration time (DIT) of 16 s. All the experiments were performed in triplicate.

#### 3.3.4. MTT Protocol

For the MTT colorimetric assays, the UM-UC-3 cell were plated at a density of 1 × 10^4^ cells per well, in 96-wells culture plates for 24 h before incubation with the compounds. Next, TMPyP or phthalocyanine solutions were added to the cells (100 µL per well) and incubated for 48 h. After this period of time, the medium was removed, the cells were washed with a PBS solution and a MTT stock solution (3.0 mg/mL in PBS buffer) was added to each well. The plates were then wrapped in aluminum foil and incubated in the darkness at 37 °C for 4 h. The resulting purple needle-shaped crystals were dissolved by the addition of 150 µL acidic isopropanol. The absorbance was measured at 570 nm using a plate reader spectrophotometer. The percentage of absorbance for each treated sample was normalized to that of the untreated control cells.

##### Calculations

The IC_50_ values (i.e., concentration of add compound that reduces cell survival by 50%) were calculated using non-linear regression analysis, sigmoidal dose-response curves (using GraphPad Prism) as shown in the equation below:

(5) $$\mathrm{MTT}\;\mathrm{reduction}\;\left(\%\right)=Bottom+\;\frac{Top-Bottom}{1+10^{\left(\log IC_{50}-\log(\left[ligand\right])\times Hillslope\right)}}$$

Herein, Bottom represents the maximum value of response (maximum percent of MTT reduction) and Top is the minimum value of response (minimum percent of MTT reduction). The log *IC*_50_ is the log of the ligand concentration ([*ligand*]) that responses midway between top and bottom. The Hillslope is the steepness of the curve.

#### 3.3.5. Fluorescence Microscopy

For microscopic evaluation, the cells were plated in coated glass coverslips with poly-L-lysine at a density of 6 × 10^4^ cells per mL before treatment. The cells were incubated with TMPyP (30 μM), ZnPc1 (20 μM) and ZnPc4 (45 μM), during 48 h at 37 °C in the dark. Immediately after incubation, cells were washed with PBS and fixed in paraformaldehyde (PFA). The coverslips were washed before mounting with VectaSHIELD medium containing the nuclear and chromosome dye DAPI (4′,6-diamidino-2-phenylindole). Samples were imaged using a confocal microscope LSM 710, Carl Zeiss (Jena, Germany).

## 4. Conclusions

**Figure 13:**
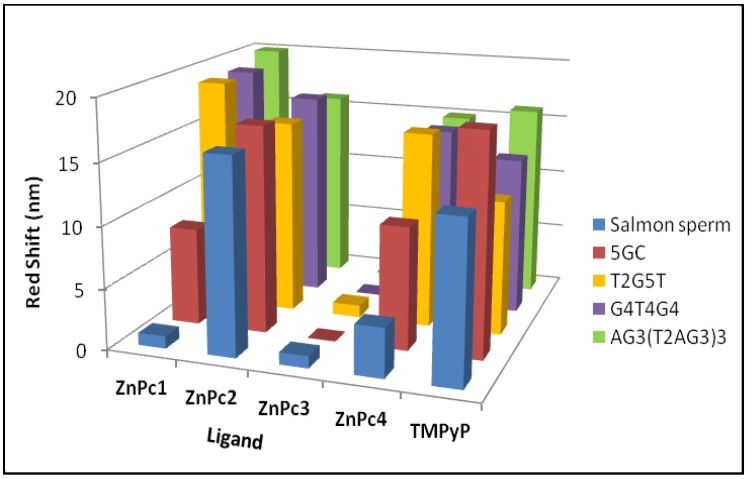
Red shifts obtained in the UV-Vis titration with the selected ligands and DNA sequences.

In summary, we have used different spectroscopic and in vitro techniques to evaluate the ability of four different cationic Pcs to interact with GQ DNA structures. The findings of these studies suggest that the two phthalocyanines, ZnPc1 and ZnPc4 have a high selectivity and affinity for G-Quadruplex over duplex structures (Figure 13).

The hyperchromic effect detected during the titration process of the phthalocyanines with different DNA topologies was discussed as being the result of the ability of GQ to prevent the aggregation of Pc. The observed hypochromic effect for PBS buffer confirms the participation of GQ in the disaggregation process.

The presence of four charges peripherally localized in ZnPc1 is responsible not only for a high affinity for GQ structures but also for a high selectivity when compared to the duplex structures; the red shift observed for duplex structure is less than half of the one observed for GQ structures. A different situation was observed for the highly charged counterpart ZnPc2, since this ligand (with the eight charges peripherally localized) shows identical affinity for all DNA topologies even for the salmon sperm.

The absence of bathochromic shifts, associated with considerable hyperchromic effect obtained for the Pc with the four positive charges less exposed, ZnPc3, are indicative of its low affinity for DNA structures. However, when this pattern is associated with a high number of charges, in ZnPc4, high affinity for GQ towards duplex structures was detected. The results obtained for TMPyP are in agreement with the literature, where the affinity for all the DNA structures was not accompanied by a high selectivity towards GQ. Taken together, these findings suggest that, ZnPc1 and ZnPc4 are superior to TMPyP in terms of high selectivity to GQ structures and ZnPc2 parallels the behavior of TMPyP.

The apparent equilibrium dissociation constants calculated for the unimolecular GQ and for the double stranded 5GC showed that the ligand ZnPc4 has the highest affinity towards the unimolecular GQ and the KD values obtained for 5GC are about 10^−4^–10^−5^ M higher than those obtained for the unimolecular GQ, pointing out to a weaker binding of the studied Pcs to double stranded 5GC.

Overall, our findings suggest that ZnPc1 and ZnPc4 have high potential as cellular nucleus targeting drugs. We demonstrated that ZnPc1 and ZnPc4 have high selectivity for G-Quadruplex DNA structures. From the obtained results some structure-activity relationship has been recognized: the existence, in the ligand structure, of a high number of positive charges results in a better affinity of the ligand to DNA structures but lacks its selectivity; the position of the positive charge is important for the interactions; a balance between the number and position of the positive charges, is a fundamental attribute for selectivity of ligands to G-Quadruplex structures. Importantly, results of in vitro assays imply that accumulation of ZnPc1 and ZnPc4 in the nucleus is probably associated with their antitumor activity. Further studies are needed to clarify the detailed binding mode of these ligands.

## Figures and Tables

**Table 1:** Sequence and topology of studied oligonucleotides.

Oligonucleotide Sequence	Topology	Abbreviation
5′-TTGGG GGT-3′	Tetramolecular G-Quadruplex	(T_2_G_5_T)_4_
5′-GGG GTT TT GGG G-3′ (*Oxytricha* repeat oligonucleotide)	Bimolecular G-Quadruplex	(G_4_T_4_G_4_)_2_
5′-AGG GTT AGG GTTAGG GTT AGGG-3′ (human telomeric repeat)	Unimolecular G-Quadruplex	AG_3_(T_2_AG_3_)_3_
5′-GCGCG CGC GC-3′	Double strand DNA	5GC
Long single strand	Double strand DNA	Salmon sperm DNA

**Table 2:** Red shifts and hypochromic/hyperchromic percentages observed when ligands were titrated with the different DNA structures.

Entry		G-Quadruplexes			Duplexes		Ligand
		(T_2_G_5_T)_4_	(G_4_T_4_G_4_)_2_	AG_3_ (T_2_AG_3_)_3_	5 GC	Salmon Sperm	
	Red shift (nm)	19	19	20	8	1	ZnPc1
(1)	Hypo/Hyper chromism (%)	+96	+90	+78	+37	−26	
	Red shift (nm)	16	17	16	17	17	ZnPc2
(2)	Hypo/Hyper chromism (%)	+85	+79	+82	+51	+54	
	Red shift (nm)	1	n.a.	0	0	1	ZnPc3
(3)	Hypo/Hyper chromism (%)	+90	n.a.	+65	+3	−14	
	Red shift (nm)	16	15	15	10	4	ZnPc4
(4)	Hypo/Hyper chromism (%)	+71	+65	+70	+60	−11	
	Red shift (nm)	11	13	16	18	13	TMPyP
(5)	Hypo/Hyper chromism (%)	−25	−27	−33	−29	−36	

Note: The + represents hyperchromism and − represents hypochromism. n.a—not available

**Table 3:** Apparent dissociation constants (KD) obtained from UV-Vis titrations.

KD (M)		
	AG_3_(T_2_AG_3_)_3_	5GC
ZnPc1	(1.62 ± 0.12) × 10^−7^	(1.38 ± 0.15) × 10^−4^
ZnPc2	(9.58 ± 0.09) × 10^−6^	(1.28 ± 0.17) × 10^−5^
ZnPc4	(7.61 ± 0.11) × 10^−8^	(4.74 ± 0.13) × 10^−4^

**Table 4:** DC_50_ values obtained for studied ligands in PBS at 25 °C.

	DC_50_ (μM)		
	5GC	(T_2_G_5_T)_4_	AG_3_(T_2_AG_3_)_3_
ZnPc1	1.59 ± 0.06	1.14 ± 0.12	1.17 ± 0.07
ZnPc2	1.93 ± 0.48	1.84 ± 0.03	1.95 ± 0.26
ZnPc4	1.30 ± 0.21	1.08 ± 0.08	1.04 ± 0.28
TMPyP	0.94 ± 0.20	1.17 ± 0.20	1.07 ± 0.09

**Table 5:** CD melting temperatures (*n* = 3).

	*T*_m_ (°C)	Δ*T*_m_ (°C)
AG_3_(T_2_AG_3_)_3_	56.1 ± 1.4	---
AG_3_(T_2_AG_3_)_3_ + ZnPc1	65.9 ± 2.1	9.8
AG_3_(T_2_AG_3_)_3_ + ZnPc4	67.9 ± 2.4	11.8
AG_3_(T_2_AG_3_)_3_ + TMPyP	63.1 ± 1.9	7.0

**Table 6:** IC_50_ values of ZnPc1, ZnPc4 and TMPyP in UM-UC3 bladder cells cells.

Ligand	IC_50_ (μM)
ZnPc1	29.44 ± 1.47
ZnPc4	58.09 ± 1.76
TMPyP	43.48 ± 1.64

## Supplementary Materials

The following are available online. Figure S1: Spectra obtained in the titrations of TMPyP with GQ and duplex DNA sequences, Figure S2. CD spectra obtained for GQ unimolecular in the presence and absence of ZnPc1 and ZnPc4, Figure S3. CD spectra obtained for the duplex oligonucleotide 5GC in the presence and absence of ZnPc1 and ZnPc4, Table S1: Spectra obtained in the titrations of TMPyP with the different DNA sequences, Table S2: Band maxims for ligands in different solvents and solutions, Table S3: Obtained molar extinction coefficients and band maximum for ligands; list of abbreviations.

## Abbreviations

T	thymine
A	adenine
G	guanine
∆λ	wavelength deviation
DNA	deoxyribonucleic acid
GQ	G-Quadruplexes
Pcs	phthalocyanines
TO	thiazole orange
PBS	phosphate buffer solution
DC_50_	concentration of ligands required to decrease the fluorescence of the probe by 50%
IC_50_	concentration of the ligand required to reduce the cell viability by 50%
T_2_AG_3_	human telomeric sequence repeat—5′-TTA GGG-3′
T_2_G_5_T	tetramolecular G-quadruplex sequence—5′-TTG GGG T-3′
(G_4_T_4_G_4_)_2_	bimolecular G-quadruplex sequence—5′-GGG GTT TTG GGG-3′
AG_3_(T_2_AG_3_)_3_	unimolecular G-Quadruplex—5′-AGG GTT AGG GTTAGG GTT AGGG-3′
5GC	double strand DNA—5′-GCG CGC GCG C-3′
UV-Vis	UV-Visible spectroscopy
G4-FID	G-Quadruplex fluorescent intercalator displacement assay
CD	circular dichroism
