# Supplementary material for: Multicharged Phthalocyanines as Selective Ligands for G-Quadruplex DNA Structures
Source: Molecules. 2019 Feb 18;24(4):733. doi: 10.3390/molecules24040733 (PMC6412362; doi:10.3390/molecules24040733)
Supplement: Supplementary file 1 [file molecules-24-00733-s001.pdf]

molecules

Supplementary Materials

Multicharged Phthalocyanines as Selective Ligands for G-Quadruplex DNA Structures

Catarina I. V. Ramos [1,*], Susana P. Almeida [1], Leandro M. O. Lourenço [1], Patrícia M. R. Pereira [1,2,3], Rosa Fernandes [2,3], M. Amparo F. Faustino [1], João P. C. Tomé [4], Josué Carvalho [5], Carla Cruz [5] andM. Graça P. M. S. Neves [1,*]

1 QOPNA & LAQV-REQUIMTE, Department of Chemistry, University of Aveiro, 3810-193 Aveiro, Portugal; susana.p.almeida@ua.pt (S.P.A.); leandrolourenco@ua.pt (L.M.O.L.); ribeirop@mskcc.org (P.M.R.P.); faustino@ua.pt (M.A.F.F.)

2 Coimbra Institute for Clinical and Biomedical Research (iCBR), Faculty of Medicine, University of Coimbra, 3000-548 Coimbra, Portugal; rcfernandes@fmed.uc.pt

3 CNC.IBILI Consortium, University of Coimbra, 3000-548 Coimbra, Portugal

4 CQE & Departamento de Engenharia Química, Instituto Superior Técnico, Universidade de Lisboa, Av. Rovisco Pais, n1, 1049-001 Lisboa, Portugal; jtome@tecnico.ulisboa.pt

5 CICS-UBI—Centro de Investigação em Ciências da Saúde, Universidade da Beira Interior, Av. Infante D. Henrique, 6200-506, Covilhã, Portugal; josueocarvalho@gmail.com (J.C.); carlacruz@fcsaude.ubi.pt (C.C.)

* Correspondence: c.ramos@ua.pt (C.I.V.R.); gneves@ua.pt (M.G.P.M.S.N.);

1.1 UV-Vis

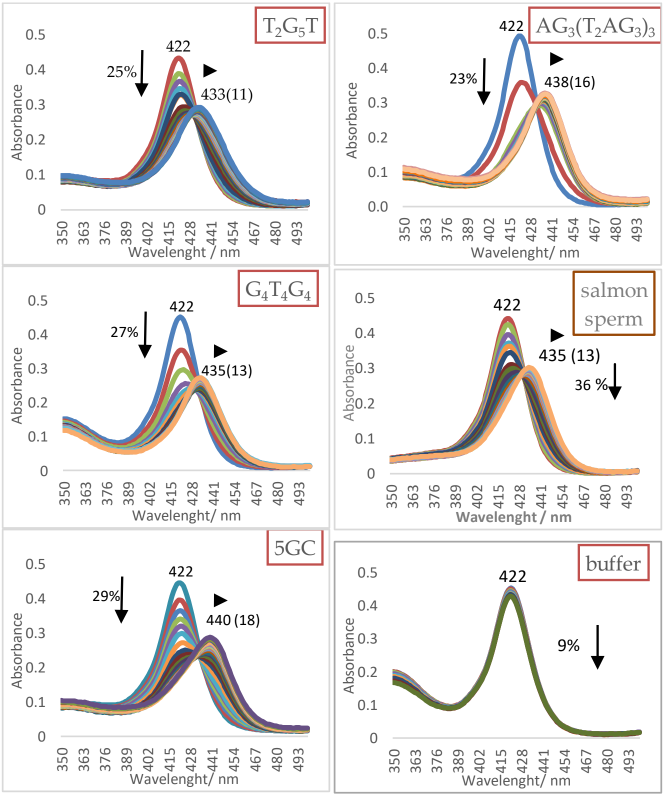

Figure S1. – UV-Vis spectra obtained in the titrations of **TMPyP** with GQ and duplex DNA sequences in PBS.

Table S1. – Spectroscopic data obtained in the titrations of **TMPyP** with different DNA sequences.

TMPyP	Hypochromism/ Hyperchromism (%)	Bathochromism (nm)
**(T_2G_5T)**	- 25	11
**($G_4T_4G_4$)$_2$**	- 27	13
**$AG_3(T_2AG_3)_3$**	- 23	16
**5GC**	- 29	18
**Salmon sperm**	- 36	13
**PBS buffer**	- 9	0

Table S2. - Band maxima for **ZnPcs1-4** in PBS and DMSO solutions.

Ligand	Band maximum in PBS (nm)	Band maximum in DMSO (nm)	Band maxima in PBS at the end of the titrations (nm)*
**ZnPc1**	672	685	691/692
**ZnPc2**	690	702	706/707
**ZnPc3**	685	686	686
**ZnPc4**	677	683	692/693

* The presented value depends on the DNA structure studied.

Table S3. – Name, molar extinction coefficients and band maxima for **ZnPcs1-4** and **TMPyP** in PBS.

Ligand	Name	Molar extinction coefficients	Band maxima (nm)
**ZnPc1**	2,9(10),16(17),23(24)-tetrakis(4-pyridylsulphanyl) phthalocyaninatozinc(II)	78217	638 / 672
**ZnPc2**	2,3,9,10,16,17,23,24-Octakis(4-pyridylsulphanyl) phthalocyaninatozinc(II)	70808	658 /690
**ZnPc3**	2,9(10),16(17),23(24)-Tetrakis(4-methoxypyridinium-1-yl) phthalocyaninatozinc(II)	23956	628 / 685
**ZnPc4**	2,3,9,10,16,17,23,24-Octakis(4-methoxypyridinium-1-yl) phthalocyaninatozinc(II)	106299	636 / 677
**TMPyP**	5,10,15,20-tetrakis(N-methylpyridinium-4-yl)porphyrin	226000	422

1.2 Fluorimetric Titrations

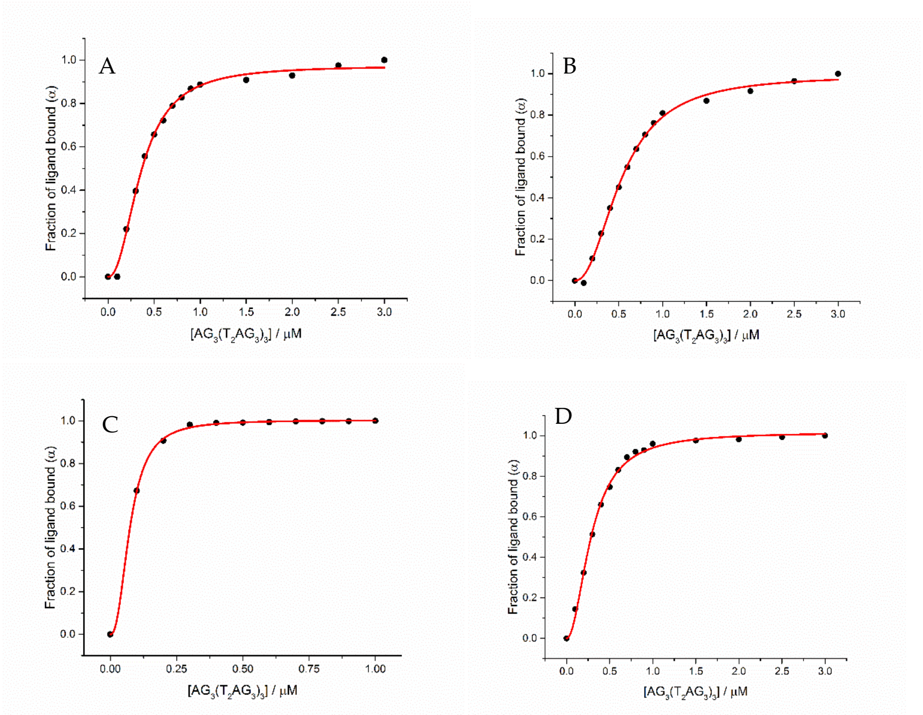

Figure S2. Saturation binding plots of ligands A) **ZnPc1**, B) **ZnPc2**, C) **ZnPc4** and D) **TMPyP** in the presence of increasing concentrations of unimolecular GQ AG3(T2AG3)3 and fitted to Hill binding equation (red curve).

1.3 Circular Dichroism

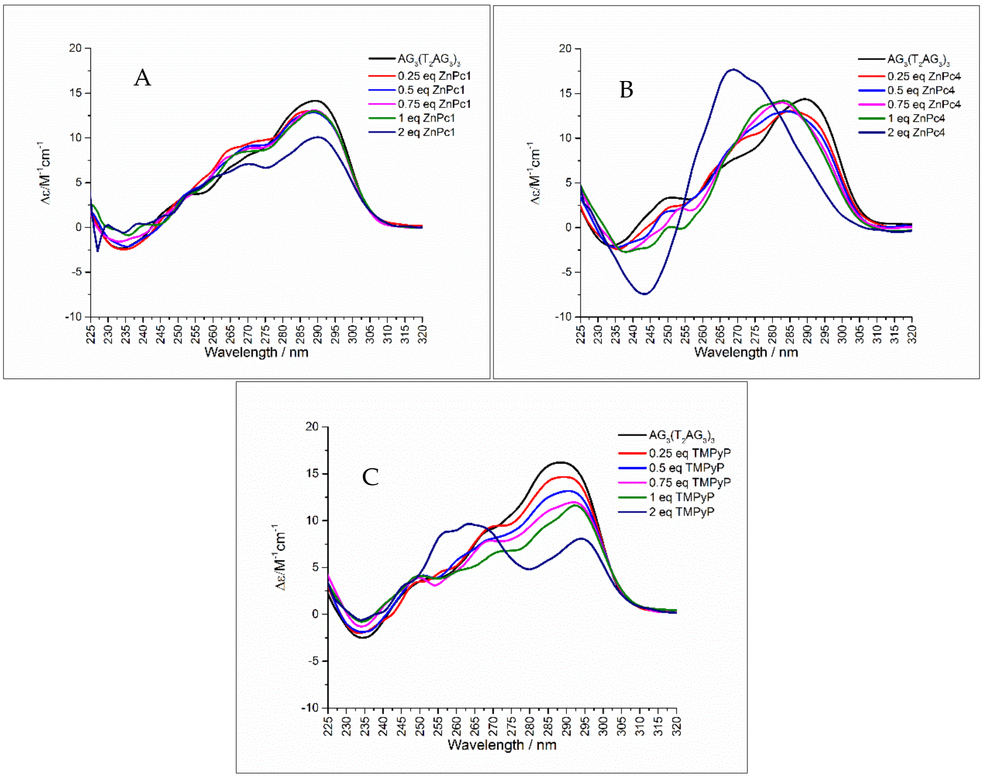

Figure S3. CD spectra obtained for unimolecular GQ AG3(T2AG3)3 in the presence and absence of A) **ZnPc1**, B) **ZnPc4** and C) **TMPyP**.

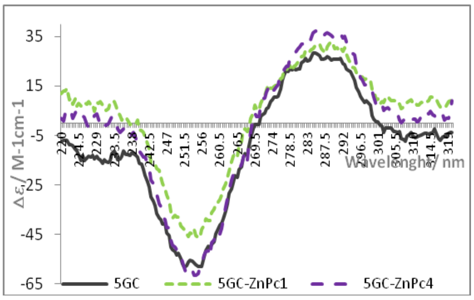

Figure S4. CD spectra obtained for the duplex oligonucleotide 5GC in the presence and absence of **ZnPc1** and **ZnPc4**.

1.4 List of abbreviations

T - thymine; **A** - adenine; **G** - guanine; **Δλ** - wavelenght deviation

DNA - deoxyribonucleic acid; **GQ** - G-Quadruplexes; **Pcs** – phthalocyanines;

TO - thiazole orange; **PBS** – phosphate buffer solution

DC50 - concentration of ligands required to decrease the fluorescence of the probe by 50%

IC50 - concentration of the ligand required to reduce the cell viability by 50%

$\mathbf{T_2AG_3}$ - human telomeric sequence repeat –5´- TTA GGG-3´

$\mathbf{T_2G_5T}$ - tetramolecular G-quadruplex sequence - 5´-TTG GGG T-3´

$\mathbf{(G_4T_4G_4)_2}$ - bimolecular G-quadruplex sequence - 5'-GGG GTT TTG GGG-3'

$\mathbf{AG_3(T_2AG_3)_3}$ - unimolecular G-Quadruplex - 5'-AGG GTT AGG GTTAGG GTT AGGG-3'

5GC - double strand DNA - 5'-GCG CGC GCG C-3'

UV-Vis - UV-Visible spectroscopy; **G4-FID** - G-Quadruplex fluorescent intercalator displacement assay; **CD** - circular dichroism
